# Thromboembolic events in COVID-19 ambulatory patients: An observational study about incidence, and thromboprophylaxis outcomes

**DOI:** 10.1371/journal.pone.0270195

**Published:** 2022-08-04

**Authors:** Rania Hammami, Jihen Jdidi, Olfa Chakroun, Fadhila Issaoui, Nouha Ktata, Hanen Maamri, Mouna Baklouti, Amine Bahloul, Rania Gargouri, Abdennour Nasri, Sameh Msaad, Samy Kammoun, Samir Kammoun, Imen Ben Rejab, Selma Charfeddine, Leila Abid

**Affiliations:** 1 Cardiology Department, Hedi Chaker Hospital, University of Medicine, Sfax University, Sfax, Tunisia; 2 Epidemiology Department Hedi Chaker Hospital, University of Medicine, Sfax University, Sfax, Tunisia; 3 Emergency Department, University Hospital Habib Bourguiba Sfax, University of Medicine, Sfax University, Sfax, Tunisia; 4 Pneumology department, Hedi Chaker Hospital, University of Medicine, Sfax University, Sfax, Tunisia; 5 Emergency Departement, Gabes Hospital, Gabes, Tunisia; Ohio State University, UNITED STATES

## Abstract

**Introduction:**

There are no clear data about the incidence and the prophylactic strategies of arterial and venous thromboembolic events (TE) in COVID-19 ambulatory patients. Thus, we conducted this study to analyze thromboembolic complications in this setting and to assess thromboprophylaxis management and outcomes in the real life.

**Patients and methods:**

This is an observational study including Covid-19 ambulatory patients. We assessed incidence of venous and arterial TE events as well as thromboprophylaxis outcomes and hemorrhagic complications. We defined high risk thrombo-embolic factor according to the Belgian guidelines which are the only guidelines that described thromboprophylaxis in COVID-19 ambulatory patients.

**Results:**

We included 2089 patients with a mean age of 43±16 years. The incidence of 30 days venous and arterial TE complications in our cohort was 1%. Venous thromboembolic complications occurred in 0.8% and arterial thromboembolic complications occurred in 0.3%.We noted at least one high-risk TE factor in 18.5% of patients but thromboprophylaxis was prescribed in 22.5% of the cases, LMWH in 18.1%, and Rivaroxaban in 3.7%. Hemorrhagic events occurred in eight patients (0.3%): five patients showed minor hemorrhagic events and three patients showed major ones (0.14%).

**Conclusions:**

Our study showed that the incidence of thromboembolic complications is very low in COVID-19 ambulatory patients. Paradoxically, there is an over prescription of thrombo-prophylaxis in this population.

## Introduction

COVID-19 is responsible for a new worldwide pandemic and has been associated with a high risk of both venous and arterial thrombotic complications, especially in hospitalized patients; the incidence of these complications seems to be correlated to the severity of the clinical presentation [1]. Several publications have reported high incidence among patients admitted to an intensive care unit (ICU), ranging from 20 to 40%, despite the administration of thromboprophylaxis [2–5]. That is why prophylactic anticoagulation is systematically recommended for COVID-19 hospitalized patients. However, according to several COVID-19 registries, 70–80% of patients showed a mild or an asymptomatic clinical form and have been treated outside the hospital [6, 7], and we don’t have enough scientific data about the thromboembolic (TE) risk in this setting. Some authors have reported an incidence of TE events ranging between 0 and 1% for COVID-19 ambulatory patients [1, 4, 8]. Many guidelines and consensus related to TE risk prevention and management in COVID-19 patients have been published by several organizations, last year. However, only the Belgian and the VAS guidelines included instructions about the management of the outpatient setting and recommended thromboprophylaxis prescription in selected patients. The VAS guidelines recommended the use of the IMPROVE score to assess the risk in ambulatory patients [9] whereas the Belgian guidelines identified many high-risk factors and recommended the prescription of thromboprophylaxis in patients with at least one high-risk factor [10]. Therefore, we conducted this study with the aim to determine the incidence of TE complications in COVID-19 outpatient people and to evaluate the real-life thromboprophylaxis management and outcomes.

## Patients and methods

We conducted a retrospective observational study in our governorate, including ambulatory patients diagnosed with COVID-19 and managed by public and private doctors, between 1^st^ October 2020 and 31^st^ December 2020. The patient will be included at least 1 month after Covid 19 infection.

We included patients with a Positive test polymerase chain reaction [PCR] for SARS-CoV-2 Obtained by nasal swab and positive rapid test. CT values < 29 are considered strong positive reactions, CT values of 30–37 are positive reactions indicative of moderate amounts of target nucleic acid and CTs of 38–40 are weak reactions indicative of minimal amounts. These patients were recorded in the regional COVID-19 registry collected by the Regional Health Directorate. We called the selected patients, or their relatives in case of death, to collect the clinical data, the drugs received and the subsequent evolution. If we failed to contact the patient, a new drawing will be performed. A prior approval of the protocol of the study was obtained from the Regional Ethics Committee, CPP SUD (Committee of Person Protection). (Reference: 0293/2021)

### Population study and data collection

Given the epidemical conditions, we avoided summoning patients to consultation; instead, we chose to call them, or their relatives–in case the patient was deceased. We conducted a telephonic questionnaire about the demographic data, medications, and follow-up. All patients or relatives gave oral informed consent when contacted.

### Inclusion criteria

We included the selected patients aged 18 years or older with a positive test for SARS-CoV-2 (PCR or rapid test) and treated outside the hospital during the period of the study. The COVID-19 positive diagnosis was confirmed by a locally obtained viral diagnostic test (polymerase chain reaction [PCR]). The initial treatment plan does not include hospitalization.

### Non-inclusion criteria

**Fig 1:**
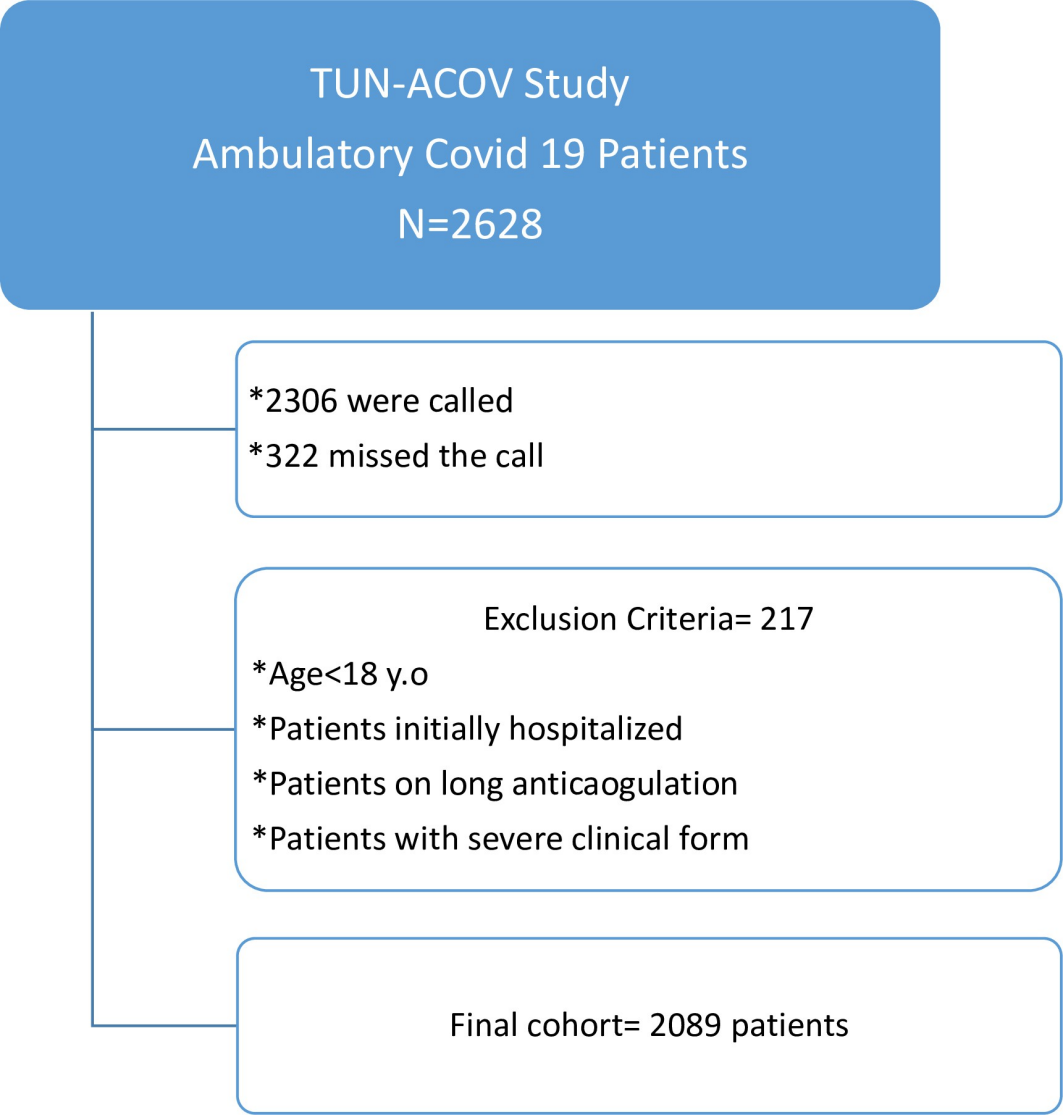
The flowchart of the study.

We did not include patients who were supposed to be hospitalized when the diagnosis of COVID-19 was made but they refused to do so; those on chronic anticoagulation; those who refused to participate in the study; and those who missed our call. The flowchart methodology of this research is available in Fig 1.

The enrollment began in January 2021; three clinical researchers (3 female, Md, who are working in the epidemiology department of Hedi Chaker hospital and who are informed about the goal of the study, without any relationship with participants) called patients and filled the telephonic questionnaire. They used the phone of the hospital. The responsible of scientific data collection controlled the word of the clinical researchers, some transcripts were returned to researchers for correction. A repeat interview was done in few patients because of lacking data, we called the doctors to check missing data in some cases. The electronic field notes (google forms) were made during the interview. The interview lasted about 10 min; the researchers finished the interview where there are no missing data.

Patient demographics and baseline clinical characteristics were recorded, including age, sex, Body mass index (BMI), cardiovascular diseases and risk factors, COVID-19 clinical presentation, D-dimer testing, disease-specific management, concomitant medications, thromboprophylaxis, and 30-day outcomes. We defined obesity by a BMI > 30Kg/m^2^ and regular physical activities by walking, jogging, or other activities for at least 30 min, 3 days a week.

We classified the clinical presentation according to the NIH guidelines [11]. *Asymptomatic or Pre-symptomatic Infection*: Individuals who test positive for SARS-CoV-2 using a virologic test but who have no symptoms that are consistent with COVID-19. *Mild Illness*: Individuals who have any of the various signs and symptoms of COVID-19 (e.g., fever, cough, sore throat, malaise, headache, muscle pain, nausea, vomiting, diarrhea, loss of taste and smell) but who do not have shortness of breath, dyspnea, or abnormal chest imaging. *Moderate Illness*: Individuals who show evidence of lower respiratory disease during clinical assessment or imaging and who have an oxygen saturation (SpO2) ≥94% on room air at sea level. *Severe Illness*: Individuals who have SpO2 <94% on room air at sea level, a ratio of arterial partial pressure of oxygen to fraction of inspired oxygen (PaO2/FiO2) <300 mm Hg, respiratory frequency >30 breaths/min, or lung infiltrates >50%.

We relieved the initial anticoagulation received by the patients when the diagnosis of Covid-19 was made. The prophylactic regimen of LMWH was defined by enoxaparin 4000U OD or BID if BMI ≥40kg/m^2^. The therapeutic regimen was defined by 1mg/kg BID with adjustment to renal function. In the other cases, we considered that the patient received an intermediate regimen [12].

Rivaroxaban was the only prescribed DOAC as it was the only one available in our country at that period. The prophylactic regimen was defined by Rivaroxaban 10 mg/d: the therapeutic regimen, by rivaroxaban 20mg /d, or 15 mg/d in case of renal failure.

We assessed the thromboembolic risk in our patients using the IMPROVE score. We considered that the patient is at high TE risk if the IMPROVE score was ≥ 4 [10].

We assessed the use of the thromboprophylaxis in the outpatient setting according the national consensus that recommend thromboprophylaxis in patients with moderate clinical presentation or at least showed one of these high risk factors: personal history of VTE, age ≥ 70 years, known thrombophilia, active cancer, Body mass index (BMI) greater than or equal to (≥35 kg/m^2^), D-dimer greater than the upper limit of normal for local laboratory (within 2 weeks of the first day of the COVID-19 test) and major surgery in the last month.

### Endpoints

The primary endpoints are composed of symptomatic venous thromboembolism, myocardial infarction, ischemic stroke, and acute limb ischemia.

In patients who received thromboprophylaxis, we assessed bleeding complications according to the definition of the International Society on Thrombosis and Hemostasis [9]. Major Bleedings are defined by fatal bleeding and/or symptomatic bleeding in a critical area or organ, such as intracranial, intraspinal, intraocular, retroperitoneal, intra-articular or pericardial, or intramuscular with compartment syndrome and/or bleeding causing a fall in hemoglobin level of 2 g/dL (1.24 mmol/L) or more or leading to transfusion of two or more units of whole blood or red cells. All non-major bleedings were considered as minor bleeds.

### Statistical analysis

We calculated the number of subjects required based on the rate of prophylactic prescription in practice (25%). Given the lack of data in the literature, this rate was defined based on a pre-survey of 20 prescribing physicians. The minimum number of subjects required was estimated at 1800 for a risk of error of 5% and a precision of 2%.

Numbers and percentages were reported for binary and categorical variables.

## Results

We included an overall cohort study of 2089 COVID-19 ambulatory patients. The mean age of our population was 43±16 years (between 18–93 years). Women accounted for 59% of the overall cohort study. The mean body mass index was 26.46±4.5 Kg/m^2^. Cardiovascular risk factors of hypertension (12.5%), hyperlipidemia (5.3%), smoking (11.9%), obesity (19.4%), and diabetes (9.3%) were common.

The clinical presentation of COVID-19 was moderate in 254 patients (12.2%), mild in 1623 patients (77.7%), and asymptomatic in 212 patients (10.1%). Most of the patients were managed by general practitioners (GPs) (45.1%), pulmonologists (8.8%), and cardiologists (4.5%).

D-Dimer tests were performed only in 94 patients (4.5%), the median of D-Dimer was 560 ng/ml with an interquartile range = 898 ng/ml. CT scan was performed in 267 patients (12.7%) and showed COVID-19 signs in 178 patients (8.5%).

Venous and arterial TE events occurred in 1% of cases (n = 20), and all-causes 30-day mortality occurred in 0.9% (19 cases). We observed non-fatal TE complications in 14 patients, fatal TE complications in 6 patients, and death without evidence of TE complications in 13 patients. In totally, 30-day death occurred in 20 patients (1.6%).

The causes of death (n = 19) were respiratory distress syndrome in 9 patients, a pulmonary embolism in 3 patients, a myocardial infarction in one patient, an ischemic stroke in one patient and an hemorrhagic stroke in a 81 very old patient who first showed pulmonary embolism and received a curative dose of LMWH. The cause of death was unkown in two patients.

Venous thromboembolic complications occurred in 17 patients (0.8%) and consisted in thrombophlebitis (11 patients) or pulmonary embolism (6 patients). Arterial embolic complications occurred in three patients (ischemic stroke in two patients and myocardial infarction in one patient).

The occurrence of venous and arterial TE complications was significantly higher in patients older than 70 years (3.5% vs0.8%), in those with cardiac diseases (5.1% vs 0.8%), especially those suffering from ischemic cardiac diseases, in those with COVID-19 signs on CT scan (5.1% vs 0.6%) and in case of moderate clinical form compared to the others clinical presentations (3.9% vs 0.5%), patients with regular physical activity showed a lower rate of primary endpoints (0.5% vs 1.4%).

We noted that only 15 patients (0.7%) showed a high IMPROVE score (≥4) and 387 patients (18.5%) had at least one high TE risk factor: VTE history in 29 patients (1.4%), pregnancy in 24 patients (1.1%), Cancer history in 21 patients (1%), hormonal contraception in 13 patients (0.6%), recent surgery or miscarriage during the last month in 17 patients (0.8%), BMI ≥ 35 Kg/m^2^ in 84 patients (4%), age more than 70 years in 142 patients (6.8%), moderate clinical presentation in 254 patients (12.2%) and D-Dimer upon to the superior limit in 28 patients (1.2%).

Thromboprophylaxis was prescribed in 472 patients (22.5%): 78 patients (3.7%) had received only DOACs, 380 patients (18.1%) had received only LMWH, and 14 patients had received first LMWH and then switched to DOACs (0.6%). We noted that GPs and cardiologists prescribed DOACs more frequently than the other specialists did. Rivaroxaban 10 mg daily was prescribed in all patients who received DOACs. Two-hundred and fifty-six patients received a prophylactic dose of LMWH, 21 patients received a therapeutic dose and 103 received an intermediate dose of LMWH.

**Fig 2:**
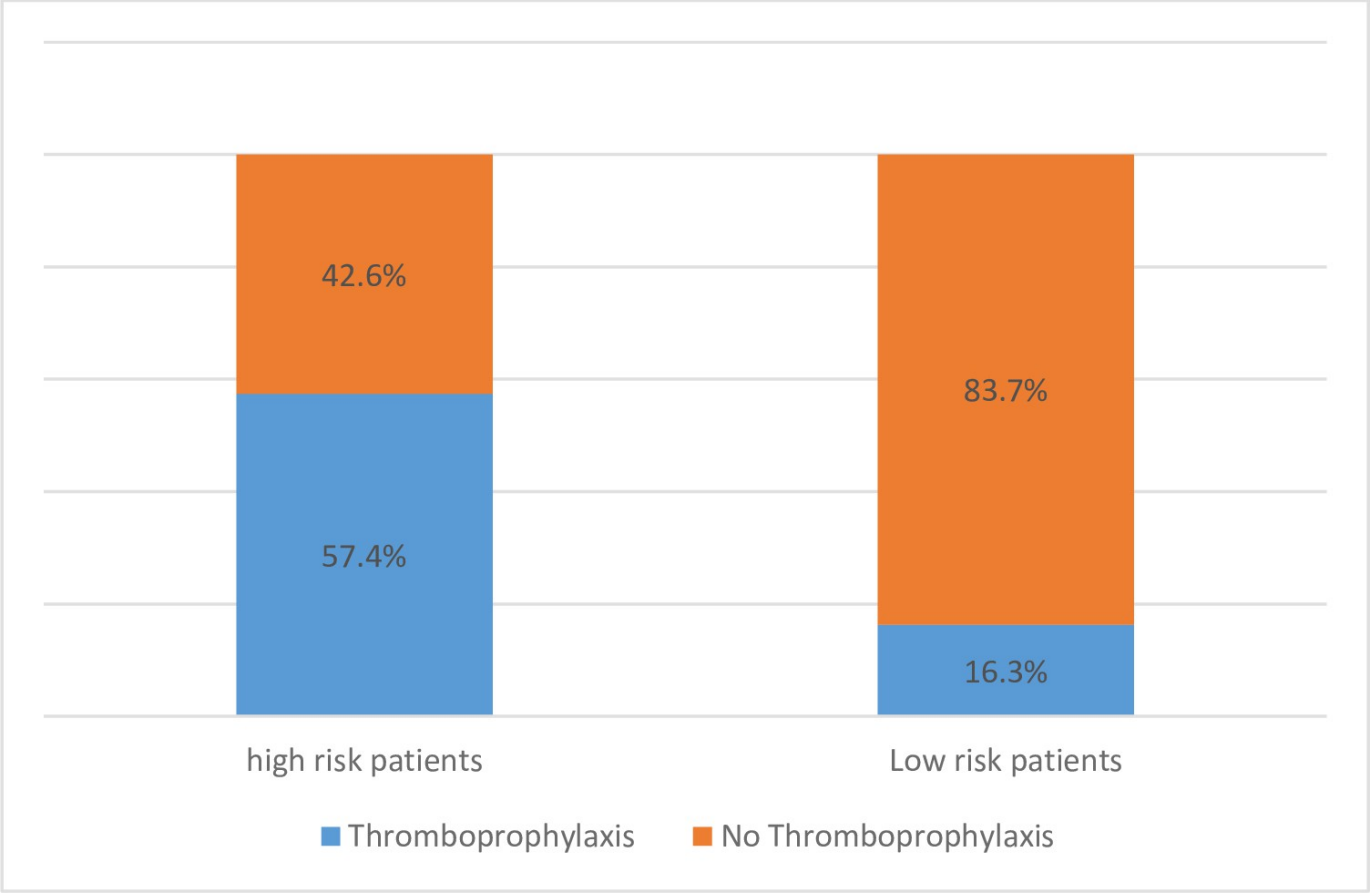
Thromboprophylaxis in patients at high risk of thromboembolic complications according to the Belgian guidelines.

Only 23.9% of our cohort showed at least one high risk TE factor. According to the Belgian guidelines, we noted an over prescription in 13.5% of patients and an under prescription in 7.9% of patients (Fig 2).

According to the IMPROVE score, only 6% of patients were considered as a high-risk patients of TE complications. An over prescription of thromboprophylaxis was noted in 19.5% patients and an under prescription in 1.6% patients.

Hemorrhagic events occurred in eight patients (0.3%): five patients showed minor hemorrhagic events and three patients showed major events (0.14%). One event occurred in a patient who received an antiplatelet inhibitor and did not receive any anticoagulant. Two events occurred in two patients who had received LMWH. One of the events ended in a fatal hemorrhagic stroke in a 72-year-old female, who was on an intermediate dose of LMWH. The second patient showed hematuria with a fall in hemoglobin level and needed a transfusion. This patient was obese (IMC = 35Kg/m^2^) and on a prophylactic dose of LMWH (enoxaparin 40mg *2), the evolution was uneventful. No patient from the Rivaroxaban group showed minor or major hemorrhagic events. All the minor events occurred in the group that received LMWH.

All the hemorrhagic complications occurred in patients who showed at least one high-risk factor of thromboembolic event and to whom the prescription of anticoagulant was well-justified.

## Discussion

Despite being a respiratory illness, COVID-19 is proved to be associated with a high risk of venous and arterial thromboembolic events. We know that the incidence of thromboembolic disease in the general population is very low, ranging between 0.1 and 0.3% annually [13], the incidence in ambulatory cancer patients is about 12.6% over 12 months of follow‐up after chemotherapy [14]. In COVID-19 patients, a recent meta-analysis of studies in COVID-19 hospitalized patients showed an overall VTE prevalence of 14.1% (95% CI, 11.6–16.9) [15]. The incidence was higher in studies that used ultrasound screening (40.3%; 95% CI, 27.0–54.3) than in those that did not (9.5%; 95% CI, 7.5–11.7). In previous randomized controlled trials conducted in non-COVID-19 hospitalized patients who received VTE prophylaxis, the incidence of symptomatic VTEs ranged between 0.3% and 1%, and the overall incidence of VTEs was between 2.8% and 5.6%.

The incidence of VTE in the COVID-19 ambulatory setting is under-described in the literature, and that is why we conducted this observational study. In the American Registry of arterial and thromboembolic complications in COVID-19 patients, there were no cases of TE events in outpatient people [1]. In a recent cohort of 220588 patients with positive SARS-Cov-2 test, the incidence of VTEs in non-hospitalized patients was 0.18% and did not differ from non-hospitalized negatively tested patients (0.22%) [16]. In our cohort, the rate of VTE events was 0.8% and was relatively high compared to the previous studies, probably because of the high rate of moderate clinical presentation (12.2%) and smoking (11.9%). In the American registry, the prevalence of moderate clinical presentation and smoking were, respectively, 9.5% and 4.1%. While in our cohort, we found that moderate clinical presentation was a strong predictor of both the TE complications and the primary safety endpoints. Besides, smoking was an independent factor of thromboembolic events especially in women.

At our knowledge, this is the first study that assess specifically management of TE events in COVID-19 ambulatory patients. Many registries did not report any case in this setting, Overstad et al reported 4 cases of venous TE in 4 non-hospitalized patients, none of these patients had major risk factors for developing VTE. The advanced cause of this complication was immobilization [17], these four patients were treated with apixaban.

Unlike COVID-19 hospitalized patients, there is a lack of recommendations and consensus about thromboprophylaxis in an outpatient setting [9, 10, 18–20]. Only two guidelines discussed the prevention of TE events in COVID-19 ambulatory patients. According to the Belgian guidelines, the use of thromboprophylaxis–especially LMWH in this setting–should be conducted in patients with high TE risk defined by at least one risk factor (known thrombophilia, personal or familial history of VTE, obesity, pregnancy, heart failure, respiratory failure, age >70 years, active cancer, and/or major surgery in the last 3 months) [10]. According to the VAS guidelines, the authors advised to calculate the IMPROVE score and to prescribe thromboprophylaxis in patients with an IMPROVE score ≥ 4 or an IMPROVE score of 2 or 3 with D-dimer greater than the upper. In fact, the IMPROVE-DD VTE score has been recently validated for the VTE risk assessment model for both non-COVID-19, and more recently, COVID-19 patients [21–23].

In our study, although only 18.5% of the cohort had at least one of the previous risk factors, and only 0.8% had a high IMPROVE score, thromboprophylaxis was prescribed in 22.5% of the cases. Thus, we noted an anticoagulant over-prescription. This could be explained by the lack of specific guidelines for the ambulatory setting and the high rate of thromboembolic complications in hospitalized patients, commonly causing death. This over-prescription was acceptable when considering the low risk of major hemorrhagic complication (0.14%) that finally occurred in patients needing an anticoagulant prescription.

The type of anticoagulant that should be prescribed in ambulatory patients was poorly debated in the literature. The disadvantages of LMWH administration in COVID-19 patients are the risk of contamination of the nurse and the need to use expensive personal protective equipment, in addition to the thrombopenia risk if prescribed for a long period. Contrariwise, the advantages of DOACs are the oral administration, the selective coagulation factor inhibition resulting in no thrombopenia, and its daily low cost. In the VAS guidelines, we suggested using in outpatient settings, either Rivaroxaban or Betrixaban, basing only on previous findings in non-COVID- 19 patient studies. In the MEGALLAN and the RECORD-4 studies, Rivaroxaban has shown benefit in reducing the risk of venous thromboembolic events among medically ill patients with elevated thrombotic risk and low bleeding risk, including those with pneumonia and sepsis. The Rivaroxaban was initiated in-hospital and continued during the post-hospital discharge period [23, 24]. COVID-19 ambulatory patients may represent a similar medically ill population that could benefit from thromboprophylaxis with Rivaroxaban. In fact, Factor Xa (FXa) plays a pivotal role in the coagulation cascade and the thrombosis physiopathology, thus, constituting an important target for anticoagulant therapy. Beyond the role in maintaining hemostasis, FXa had also been linked to inflammation via protease-activated receptors (PAR-1 and PAR-2), leading to pleiotropic effects during chronic disorders, such as atherosclerosis, inflammation, and neovascularization. Therefore, by inhibiting Factor Xa-induced cell activation, rivaroxaban could also prevent the progression of various pathological conditions [25].

In COVID-19 patients, the other proposed therapeutic mechanism of factor Xa inhibitor on CoV cellular infection is that SARS coronavirus binds to the host cell expressing a receptor of the coronavirus spike protein. Factor Xa then acts as a proteolytic enzyme, cleaving the spike protein into spike protein 1 and spike protein 2. Spike protein 1 then parts from the virus-cell complex with or without the attached receptor, while spike protein 2 serves to aid in the fusion of the virus and cell membranes. The addition of a factor Xa inhibitor (FXa) blocks FXa from acting as a proteolytic enzyme, therefore, leaving the spike protein intact, preventing the virus and host cell membrane fusion [26]. Based on the potential role of factor Xa in the pathogenesis of coronavirus morbidity and mortality, Rivaroxaban may prevent severe COVID-19.

Recently, the results of ACTIV-4b trial have been published which is a randomized trial that assessed whether anticoagulant or antiplatelet therapy can safely reduce major adverse cardiopulmonary outcomes among symptomatic but clinically stable outpatients with COVID-19. The main result was that among this population, treatment with aspirin or apixaban (5mg or 10 mg) compared with placebo did not reduce the rate of a composite clinical outcome, the study was terminated after enrollment of 9% of participants because of an event rate lower than anticipated [27]. The adjudicated primary composite end point (a composite of all-cause mortality, symptomatic venous or arterial thromboembolism, myocardial infarction, stroke, or hospitalization for cardiovascular or pulmonary cause) occurred in 1 patient (0.7%) in the aspirin group, 1 patient (0.7%) in the 2.5-mg apixaban group, 2 patients (1.4%) in the5-mg apixaban group, and 1 patient (0.7%) in the placebo group. These findings are in accordance with our study.

### Study limitations

The current study must also be interpreted within the limit and we probably underestimated the incidence of VTEs as we assessed only symptomatic VTE events, as there was not any Doppler screening. Besides, it is a descriptive study so conclusions cannot be generalized to the target population.

## Conclusions

Thromboembolic complications in COVID-19 ambulatory patients are low. Our observational study showed that there is a trend to an over-prescription of thromboprophylaxis in real practice especially with the easy availability of NOAC.

## Supporting information

S1 Checklist COREQ (COnsolidated criteria for REporting Qualitative research) checklist. (PDF)

S1 Data The data of the series. (XLSX)

S1 File The google form of the interview guide: https://docs.google.com/forms/d/1iYwaL2uFGTZC21RLvZa6gPVpqJDD4YOyNOibxpq9H8I/edit. (PDF)
