# Supplementary material for: Thromboembolic events in COVID-19 ambulatory patients: An observational study about incidence, and thromboprophylaxis outcomes
Source: PLoS One. 2022 Aug 4;17(8):e0270195. doi: 10.1371/journal.pone.0270195 (PMC9352084; doi:10.1371/journal.pone.0270195)
Supplement: S1 File — (PDF) [file pone.0270195.s003.pdf]

Covid19 (real—life practice)

Questionary for COVID-19 patients

*Obligatoire

1. Name and pronoun *

2. Age (years) *

3. sex *

Une seule réponse possible.

Male

female

4. Wheight (in Kg) *

5. Size (in cm) *

6. Profession *

.

Stduent

without profession

Retraited

sedentary

physical activity

Doctor

nurse

Autre :

when you were labeled covid + *

you were managed in full at home you

were hospitalized immediately

you were managed at home at the beginning and then hospitalized secondarily

PCR+ date (approximately) *

Habits *

No

Weaned smoker over 2 years old

substance

abuse

Other:

If smoking, specify the number in pack year (in PA)

Do you have regular physical activity during the last 6 months (At least 30 min, three times a week)

never

someti
mes

always

on estrogen-positive contraception *

yes

no

not concerned (male)

Medical history *

- [ ] None
- [ ] High blood pressure
- [ ] Dyslipidemia
- [ ] Stroke ATCD (stroke) Anemie
- [ ] Cancer in remission
- [ ] Active hypothyroid
- [ ] hyperthyroidism
- [ ] cancer
- [ ] Chronic renal failure (not dialysis) chronic
- [ ] dialysis
- [ ] Adrenal insufficiency
- [ ] Hepathopathy
- [ ] system disease
- [ ] neuromuscular disease (multiple sclerosis, myopathy, myasthenia. ......)
- [ ] Diabete
- [ ] Others ______________________

:

if hypertensive

- [ ] blood pressure not measured during the period covid
- [ ] blood pressure measured during covid by you same
- [ ] blood pressure measured by a doctor
- [ ] lower or eqgal balanced voltage 140/90
- [ ] you have changed your antihypertensive treatment
- [ ] you have kept the same antihypertensive treatment

15. History of heart disease*

.

- [ ] None
- [ ] Atrial fibrillation
- [ ] Ischemic heart disease
- [ ] Mechanical prosthesis
- [ ] Valvulopathies Heart
- [ ] failure
- [ ] Other : ____________________

ATCD of respiratory disease *

- [ ] No
- [ ] COPD (chronic bronchopneumoapathy)
- [ ] asthma
- [ ] dilation of the bronchi
- [ ] pulmonary fibrosis
- [ ] sleep apnea syndrome
- [ ] Others : ____________________

Other antecedents *

- [ ] None
- [ ] Abortion < 1 month
- [ ] delivery <1 month
- [ ] Orthopedic, gynecological, urological, carcinological surgery «1 month Pregnancy in
- [ ] progress (when she discovered covid)
- [ ] contraceptives
- [ ] Other : ____________________

16. History of thromboembolic complications

yes

No

ATCD of hemorrhagic complications *

- [ ] No
- [ ] Intracerebral hemorrhage
- [ ] Gastrointestinal hemorrhage
- [ ] Gynecological hemorrhage
- [ ] Urological hemorrhage
- [ ] parietal hematoma or lesse, soft parts
- [ ] Other : ______

sick on anticoagulants at long course *

non

sintrom

previscan

rivaroxaban (Riva or Xaban)

LMWH at long course

Other : ______

Sick on anti-active ingredients *

None

Clopidogrel (plavix, pidogrel, clopix, cloven, copigrel)

Ticagrelor (Brilique)

Aspegic or Kardegic or aspirin

Taking Aspégic for the first time during covid *

no

yes

Other treatments at Iong course *

No chemotherapy

non-invasive ventilation at home

immunosuppressant corticosteroid

therapy

Other : ______________________

Clinical form of covid 19 *

Asymptomatic

Mild (diarrhea , fever , anosmia............) without dyspnea

Moderate (respiratory signs, dyspnea with oxygen need , but O2 needs <4l/min)

severe (need for hospitalization from the outset)

Duration of symptoms in days *

____

Chest CT *

unrealized

realized and

normal

realized and pathological

did you do a dosage of DDimères *

OUÎ

No

the highest level dosed in ng /mI exp the normal threshold < 500 ng/ml

____

Prescribing physician

Private general practitioner

General practitioner public

health Private cardiologist

Hospital cardiologist

Private pulmonologist

Hospital pulmonologist

Self-prescription

Other : ______________________________

17. The patient received anticoagulation *

	no	an injection /j (enoxa 40)	two injections/ d (enoxa 40*2/d)	two injections /J (curative: enoxa weight *2/j)	Rivaroxaban 10	Rivaroxaban 15	Rivarox 2C
TTT in first intension							
TTT in 2nd intension							
TTT in 3rd intension							

Mode of Injection of LMWH

self injection

neighbourhood or service nurse

member of the family

Pharmacy officinale

Other : ______________________

duration LMWH (in days)

____

duration Rivaroxaban (in days)

____

total duration of anticoagulant TTT (in day)

____

1. Rest of the Treatment received *

- [ ] No
- [ ] Antibiotics
- [ ] Vitamin supplements Oxygenotherapie
- [ ] at home
- [ ] Systemic corticosteroids (oral or injected)
- [ ] analgesic (paracetamol)
- [ ] Stopping
- [ ] contraceptives Other
:

Follow-up *

- [ ] Favourable developments
- [ ] Thromboembolic complications
- [ ] need for hospitalization respiratory
- [ ] worsening Hemorrhagic
- [ ] complications Neurological
- [ ] complications renal complications
- [ ] Death
- [ ] Other : ______________________________

cause of death

____

if thromboembolic complications, specify the Type

- [ ] thrombophlebitis
- [ ] pulmonary
- [ ] embolism Stroke
- [ ] IDM
- [ ] ischemia of the member
- [ ] mesenteric infarction
- [ ] fistula thrombosis in dialysis patients
- [ ] Other : ______________________

1. if thromboembolic complication, specify the time (in days) from the onset of symptoms

____

1. Hemorrhagic complications on anticoagulants *

No

digestive hemorrhage

intracerebral hemorrhage

urological hemorrhage

hematoma of soft parts

minimal bleeding (epistaxis, gingivorragia, ...)

41. post covid syndrome (after the guerrison you have kept symptoms)*

- [ ] cephalea
- [ ] palpitations chest
- [ ] pain dyspnea
- [ ] cough
- [ ] fatigue
- [ ] sleep disorders loss of
- [ ] taste (agueusia)
- [ ] loss of smell (anosmia)
- [ ] difficulty concentrating
- [ ] myalgia
- [ ] abdominal pain
- [ ] none
- [ ] Other : ______________________

1. have you consulted for this postcovid syndrome *

yes

No

1. You have made explorations for this postcovid syndrome *

- [ ] No
- [ ] ECG
- [ ] Echocoeur
- [ ] Echoabdominal
- [ ] Thraocic Troponin
- [ ] Scanner
- [ ] BNP
- [ ] Ddimers
- [ ] Rhythmic Holter
- [ ] Other : ______________________

1. Results of these examinations

no examination carried

out and normal all

Other : ____________________

1. comments (name of lïnvestigateur)

____
